# Flexible and Stretchable Liquid Metal Electrodes Working at Sub-Zero Temperature and Their Applications

**DOI:** 10.3390/ma14154313

**Published:** 2021-08-02

**Authors:** Peng Xiao, Ju-Hyung Kim, Soonmin Seo

**Affiliations:** 1 Department of Bionano Technology, Gachon University, Seongnam 13120, Korea; zhongpengxiao@gmail.com; 2 Department of Chemical Engineering, Ajou University, Suwon 16499, Korea; 3 Department of Energy Systems Research, Ajou University, Suwon 16499, Korea

**Keywords:** liquid metals, gallium alloys, Galinstan, flexible electronics photodetectors, solar-blind photodetection

## Abstract

We investigated characteristics of highly flexible and stretchable electrodes consisting of Galinstan (i.e., a gallium-based liquid metal alloy) under various conditions including sub-zero temperature (i.e., <0 °C) and demonstrated solar-blind photodetection via the spontaneous oxidation of Galinstan. For this work, a simple and rapid method was introduced to fabricate the Galinstan electrodes with precise patterns and to exfoliate their surface oxide layers. Thin conductive films possessing flexibility and stretchability can be easily prepared on flexible substrates with large areas through compression of a dried suspension of Galinstan microdroplets. Furthermore, a laser marking machine was employed to facilitate patterning of the Galinstan films at a high resolution of 20 μm. The patterned Galinstan films were used as flexible and stretchable electrodes. The electrical conductivity of these electrodes was measured to be ~1.3 × 10^6^ S m^−1^, which were still electrically conductive even if the stretching ratio increased up to 130% below 0 °C. In addition, the surface oxide (i.e., Ga_2_O_3_) layers possessing photo-responsive properties were spontaneously formed on the Galinstan surfaces under ambient conditions, which could be solely exfoliated using elastomeric stamps. By combining Galinstan and its surface oxide layers, solar-blind photodetectors were successfully fabricated on flexible substrates, exhibiting a distinct increase of up to 14.7% in output current under deep ultraviolet irradiation (254 nm wavelength) with an extremely low light intensity of 0.1 mW cm^−2^, whereas no significant change was observed under visible light irradiation.

## 1. Introduction

Recently, liquid metals based on gallium (Ga) alloys have received increasing attention, owing to their outstanding electrical and mechanical properties [1,2,3]. The Ga-based metal alloys that exist as virtually non-toxic liquids at room temperature show not only excellent stretchability and deformability but also environmental friendliness and recyclability. In this context, extensive research efforts have been devoted to the development of various applications using Ga-based metal alloys, such as sensors [4], reconfigurable antennas [5], and soft electrodes [6]. These fluidic metal alloys also show great potential for electronic skins [7,8] and wearable electronics [9,10,11]. Among various Ga-based metal alloys, Galinstan (68.5% Ga, 21.5% indium (In), and 10% tin (Sn)) has been notably studied in recent years due to its remarkably low toxicity and melting point (~−19 °C) [12], which is also suitable for flexible and stretchable devices operating below 0 °C compared to other eutectic gallium indium (EGaIn).

Although Galinstan shows outstanding properties including flexibility and stretchability even under cold conditions, its high surface tension and rapid oxidation rate hinder the fabrication of desirable patterns for electronic devices and circuits in comparison with other functional materials [13,14,15]. Various methods for Galinstan patterning thus have been developed and enhanced, including microfluidic injection [16,17,18,19,20,21], photolithography [22,23], stencil lithography [24,25,26,27], imprint lithography [28], microcontact printing [29,30], and composite material synthesis [31]. Each of these methods has individual advantages (i.e., high processability, high resolution limits, high stability, or cost-effective fabrication); however, integrating all the advantageous elements is still a challenge. For instance, the demanding process conditions for delaying surface oxidation or preventing leakage of the liquid metal or the low patterning resolution limits still need to be improved depending on the method. Laser ablation is one of the patterning methods with high processability, enabling the rapid fabrication of electrodes with complex features [32,33]. Although the pattern resolution is limited by the beam spot of laser, which is normally in the range of tens to hundreds of micrometers, this method can be directly employed for various applications without laborious pre- and/or post-treatments.

In addition, it is worth noting that the spontaneous oxidation of Galinstan in air leads to the formation of thin Ga oxide (Ga_2_O_3_) films on Galinstan surfaces [3,34,35]. Ga_2_O_3_ with a wide bandgap (~4.9 eV), which is rapidly formed on Galinstan surfaces in less than one second during the patterning processes in air [2], is transparent in the visible light region and exhibits high light-absorption coefficients in the deep ultraviolet (UV) region [35,36,37,38]. The surface oxide layers normally degrade the metallic properties of Galinstan; however, these layers are also expected to be utilized for solar-blind photodetection (i.e., deep UV detection insensitive to solar radiation) if they can be neatly separated from the bulk material [39].

Herein, we investigated characteristics of the Galinstan electrodes to verify flexibility and stretchability under various conditions including sub-zero temperature (i.e., <0 °C). For this study, a simple and rapid method was employed to fabricate the Galinstan electrodes with precise patterns. Thin Galinstan films with high electrical conductivity were uniformly deposited on flexible polydimethylsiloxane (PDMS) substrates by the compression of Galinstan microdroplets and sequentially patterned using a fiber laser marking machine. The transparent PDMS substrates were found to be undamaged by a laser with a wavelength of 1064 nm, and only the Galinstan layers were ablated according to the designed electrode shapes. In addition, the surface oxide (i.e., Ga_2_O_3_) layers of the Galinstan electrodes were also examined to confirm their potential for solar-blind photodetection. For the photoactive components, the thin Ga_2_O_3_ films, spontaneously formed on the Galinstan surfaces, were exfoliated using elastomeric PDMS stamps [39,40] and then transferred onto the patterned Galinstan electrodes to complete the device structure for solar-blind photodetection. By combining Galinstan and Ga_2_O_3_ films, sensitive solar-blind photodetectors were successfully fabricated on flexible substrates. The photodetectors showed a distinct increase of up to ~15.1% in output current under deep UV irradiation (254 nm wavelength) with an extremely low light intensity of 0.1 mW cm^−2^, whereas no significant change was observed under visible light irradiation.

## 2. Materials and Methods 

### 2.1. Preparation of Galinstan Microdroplets

**Figure 1:**
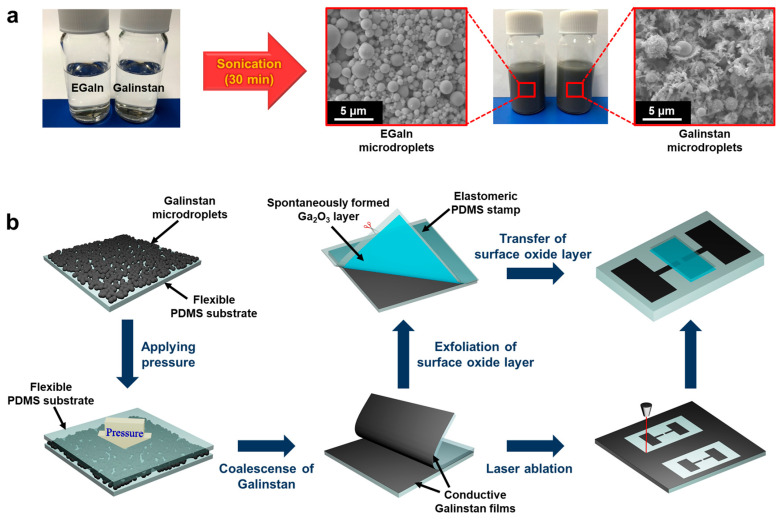
(**a**) Preparation of suspensions comprising EGaIn and Galinstan microdroplets by sonication. (**b**) Schematic illustration of the fabrication of flexible solar-blind photodetector using Galinstan microdroplets.

Galinstan (68.5 wt.% Ga, 21.5 wt.% In, and 10.0 wt.% Sn) and PDMS (SYLGARD 184) were purchased from Geratherm Medical AG (Geratal, Germany) and DOW (Midland, MI, USA), respectively. EGaIn (75.5 wt.% Ga and 24.5 wt.% In) was purchased from Sigma-Aldrich Korea (Seoul, Korea). To prepare the Galinstan microdroplets, 0.5 g of Galinstan was sonicated in ethanol for 30 min (80 W, 40 KHz). Galinstan was well dispersed during the sonication process and rapidly stabilized by surface oxidation in ethanol, resulting in the suspension of Galinstan microdroplets (<10 μm) as shown in Figure 1a. The same procedure was repeatedly performed for the preparation of the EGaIn microdroplets.

### 2.2. Preparation of Elastomeric PDMS Substrates and Stamps

For the PDMS substrates, the PDMS precursor, consisting of a silicone elastomer base and a curing agent (in a 10:1 weight ratio), was poured onto a flat Petri dish, and subsequently degassed in a vacuum desiccator for 1 h. The sample was cured at 80 °C for 1 h in a convection oven. After thermal curing, the PDMS film was easily peeled off from the Petri dish and then cut into 50 mm × 50 mm specimens. The thickness of each substrate was measured as ~1 mm. Sticky elastomeric PDMS stamps were individually prepared to exfoliate the thin Ga_2_O_3_ films. The mixing ratio of the PDMS precursor was modified to a 11:1 weight ratio to delay the saturation of cross-linking and enhance its adhesive properties, and the same preparation procedure as for the PDMS substrates was followed.

### 2.3. Fabrication of Patterned Galinstan Electrodes

The suspension containing the Galinstan microdroplets was drop-dispensed onto the flat PDMS substrate and then slowly dried at 30 °C for 24 h to avoid the formation of structural defects induced by rapid evaporation of the solvent. The dried suspension of the microdroplets in thin-film form was covered with another flat PDMS substrate and subsequently pressed at a pressure of 15 MPa for 5 s to collapse the surface oxide layers and connect Galinstan. After releasing the pressure, the upper PDMS mold was peeled off from the bottom PDMS substrate, resulting in the formation of thin conductive Galinstan films deposited on both PDMS substrates.

The Galinstan films were patterned using a laser marking machine (50 W, Dongil Laser Technology, Gwangju, Korea). The scanning speed of the laser marking machine was 600 mm s^−1^, and the power intensity was 1.0 % of its maximum power (i.e., 0.5 W). A high resolution of 20 μm was achieved in the Galinstan patterning process by this laser ablation method.

### 2.4. Characterization of Flexible and Stretchable Liquid Metal Electrodes

To investigate characteristics of the liquid metal electrodes under various conditions, thin conductive films (15 × 25 mm^2^) of Galinstan and EGaIn were individually prepared with the same procedure. The thickness of each film was 1 μm. A semiconductor characterization system (4200-SCS, Keithley, Beaverton, OR, USA) was used for the measurements.

### 2.5. Fabrication of Flexible Solar-Blind Photodetectors Using Ga_2_O_3_

The elastomeric PDMS stamp was brought into contact with the surface of the Galinstan film. A thin Ga_2_O_3_ film (<10 nm), which was spontaneously formed on the Galinstan surface, was attached to the sticky PDMS stamp and easily exfoliated from the Galinstan film by peeling off the stamp. The transparent Ga_2_O_3_ film on the PDMS stamp was cut and placed between two patterned Galinstan electrodes to complete the device structure.

### 2.6. Measurements

A semiconductor parameter analyzer (Keithley 4200, Beaverton, OR, USA) and resistivity meter (Loresta-GX MCP-T700, Mitsubishi Chemical Analytech, Yamato, Japan) were used to measure electrical properties and perform bending tests on the devices. Photocurrent measurements were performed for deep UV and visible regions using a UV lamp (8 W, Vilber Lourmat, Collégien, France) and a halogen lamp (FOK-100W, Fiber Optic Korea, Cheonan, Korea). The surface morphology was also investigated using atomic force microscopy (AFM; Nanoscope IIIa, Digital Instruments, Bresso, Italy) and scanning electron microscopy (SEM; JSM-7500F, Tokyo, Japan).

## 3. Results and Discussion

Suspensions comprising microdroplets of Galinstan and EGaIn were individually prepared as shown in Figure 1a. It exhibited a matt dark gray color due to diffuse reflections and surface oxide layers of the microdroplets. As schematically illustrated in Figure 1b, the suspension was drop-dispensed onto a flat PDMS substrate and then slowly dried at 30 °C for 24 h. As the slow drying process hindered the formation of structural defects induced by rapid evaporation of the solvent, the microdroplets were densely aggregated in thin-film form with high uniformity. For Galinstan, the size of each microdroplet was less than 5 μm, and rod-shaped particles were also observed between the rounded droplets. It is well known that rounded Galinstan microdroplets are surrounded by a thin layer of carbon and Ga_2_O_3_, of which the inner core is composed of Ga, In, and Sn [41,42]. As previously reported, the rod-shaped particles possibly consisted of Ga oxide monohydroxide ((GaO)OH) [43]. Note that Ga in Ga-based metal alloys can react with decomposed OH^−^ in the presence of O_2_, leading to the crystallization of (GaO)OH as follows:

2Ga + 2OH^−^ + O_2_ → 2(GaO)OH (1)

The amount of rod-shaped particles is significantly less than that of round particles, and it is expected to be further reduced at low-temperature conditions because the crystallization strongly depends on heat and reactive oxygen species originating from sonication [44,45].

**Figure 2:**
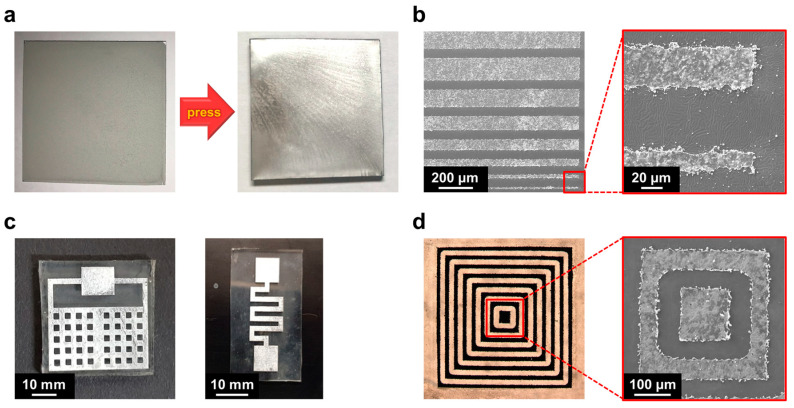
(**a**) Photographs of (left) a dried suspension of Galinstan microdroplets in thin-film form on a flexible PDMS substrate, and (right) a conductive Galinstan film fabricated by compression and separation using PDMS. (**b**) SEM images of patterned Galinstan films, of which the minimum line width is ~20 μm. (**c**) Photographs of exemplary Galinstan films, patterned by laser ablation. (**d**) Optical microscopy image and its close-up SEM image of an exemplary Galinstan structure with complex pattern.

The thin film comprising aggregated Galinstan microdroplets was not electrically conductive because each droplet was fully covered by a non-conductive Ga_2_O_3_ layer. Thus, another flat PDMS substrate for protecting Galinstan was brought into contact with the thin film, and an external pressure of 15 MPa was sequentially applied to the sample to collapse the surface Ga_2_O_3_ layers and connect Galinstan. With the collapse of the surface Ga_2_O_3_ layers, the Galinstan microdroplets were connected to achieve a continuous phase between the two PDMS substrates. After peeling off the upper PDMS substrate from the bottom PDMS substrate, thin Galinstan films were consequently formed on both PDMS substrates (see Figure 2a). The thickness of each glossy film was measured to be less than 1 μm, of which the surface partially cracked due to rapid surface oxidation during the peeling process.

A fiber laser marking machine (λ ~ 1064 nm) was employed for the direct patterning of the Galinstan films, enabling the fabrication of accurate and desirable features with sub-100 μm resolution. Various exemplary features of the patterned Galinstan films are shown in Figure 2b–d. The smallest feature size of the Galinstan line was 20 μm. Laser ablation is a fast and precise method for patterning liquid metal electrodes, facilitating the fabrication of complex and hollow patterns. In addition, this light-based patterning method does not cause significant damage to the transparent substrates, such as glass and PDMS, which do not directly absorb the energy from a fiber laser. After completing the laser ablation process, partial buckling was observed on the PDMS substrates.

**Figure 3:**
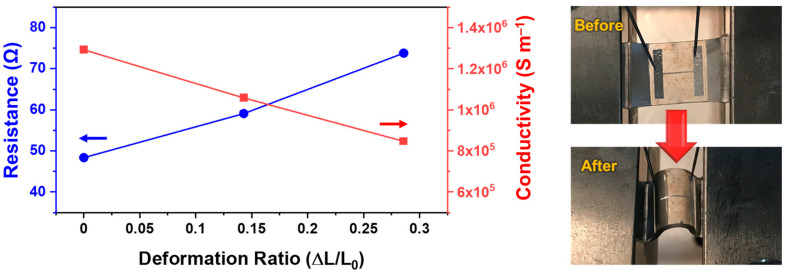
Electrical resistance and conductivity of a line-patterned Galinstan electrode (80 μm × 5 mm) corresponding to the structural deformation of the PDMS substrate.

The electrical resistance and conductivity of the Galinstan film were measured corresponding to the structural deformation of the PDMS substrate. For the measurements of electrical properties, the patterned Galinstan electrode (80 μm × 5 mm) was used as shown in Figure 3. Its thickness was less than 1 μm. The electrical resistance and conductivity were initially measured as 48.3 Ω and ~1.3 × 10^6^ S m^−1^, respectively. In response to the deformation ratio, the electrical resistance gradually increased to 73.8 Ω, corresponding to an electrical conductivity of ~8.5 × 10^5^ S m^−1^. In comparison with pure Galinstan, in which the electrical conductivity was found to be ~3.5 × 10^6^ S m^−1^, the relatively low electrical conductivity of the Galinstan films used in this work could be attributed to the partial cracks and insulating components remaining in the films, such as (GaO)OH and Ga_2_O_3_. However, since the majority of the film components were Galinstan, the fabricated films still possessed electrical conductivity high enough to be used as flexible electrodes. It should be noted that the electrical conductivity of the fabricated Galinstan film (~1.3 × 10^6^ S m^−1^) is slightly lower than that of the thin EGaIn film (~2.2 × 10^6^ S m^−1^) prepared using the same procedure [39]. It is possibly originating from the content of the insulating material in the suspension. As shown in Figure 1a, the content of the rod-shaped particles in the Galinstan suspension is significantly higher than in the EGaIn suspension under our experimental conditions, which may cause a decrease in overall electrical conductivity.

**Figure 4:**
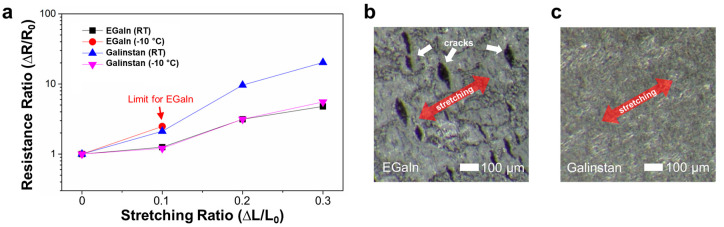
(**a**) Changes in electrical resistances of EGaIn and Galinstan films corresponding to lateral stretching at varied temperatures (i.e., room temperature (RT) and −10 °C). (**b**) SEM image of a cracked EGaIn film after lateral stretching below 0 °C. (**c**) SEM image of a stretched Galinstan film below 0 °C, exhibiting excellent film continuity.

One advantageous property of Galinstan is the liquid phase, maintaining its flexibility and stretchability, even below 0 °C. To compare with EGaIn, electrical resistances of the two materials were measured corresponding to lateral stretching (up to ~130%) of the PDMS substrates. For the measurements, the flat Galinstan and EGaIn electrodes were individually prepared on the PDMS substrates (15 × 25 × 1 mm^3^) and then stretched up to 130% at room temperature and −10 °C, respectively. Changes in the electrical resistances upon lateral stretching are shown in Figure 4a. At room temperature, the electrical resistances of both materials slightly increased with the stretching ratios, which is possibly originating from structural deformation [26]. At the temperature of −10 °C, the cracks were generated inside the EGaIn film upon the lateral stretches (see Figure 4b), leading to significant reduction in the film continuity. When the stretching ratio was above 110%, the electrical conductivity of the EGaIn film was thus not observed. However, differently from EGaIn, the Galinstan film was still electrically conductive even if the stretching ratio increased up to 130%. These results were caused by the difference in the melting points of the two materials (i.e., ~−19 °C for Galinstan and ~16 °C for EGaIn). At −10 °C, the EGaIn film in the solid phase was significantly damaged, whereas the Galinstan film in the liquid phase showed excellent film continuity (see Figure 4c).

**Figure 5:**
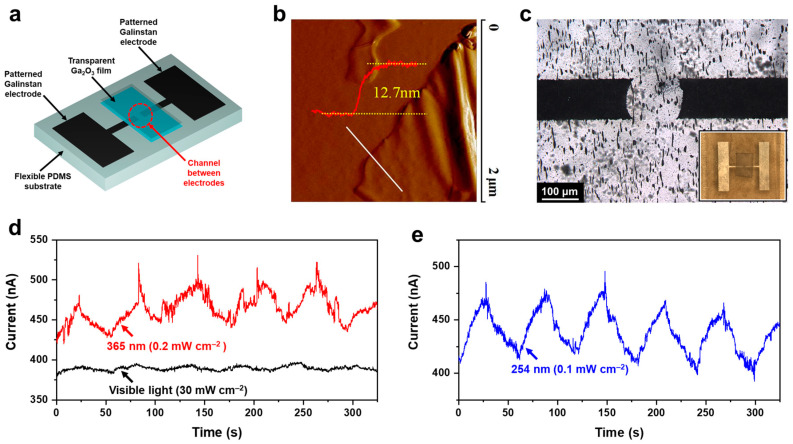
(**a**) Schematic illustration of solar-blind photodetector. (**b**) AFM image of transparent Ga_2_O_3_ film, exfoliated using a PDMS stamp. The height profile along the white line is also indicated. (**c**) Optical microscopy image of a channel between two patterned Galinstan electrodes. The black areas correspond to Galinstan beneath the transparent Ga_2_O_3_ film. Photograph of the fabricated photodetector is also shown in the inset. (**d**) Output characteristics of the solar-blind photodetector under irradiations of visible light (with a halogen lamp; ranging from 350 to 900 nm) and UV light of 365 nm wavelength, respectively. (**e**) Output characteristics of the solar-blind photodetector under deep UV irradiation (254 nm wavelength). The on/off switching of each irradiation was manually performed at 30 s intervals, and the output characteristics were constantly measured at a sample bias voltage of 0.1 V.

In addition, the Ga_2_O_3_ layer, which was spontaneously formed on the Galinstan film, was neatly exfoliated using an elastomeric PDMS stamp for further investigation. It should be noted that inherently high adhesion between the thin oxide shell and PDMS was reported [42], and the elastomeric PDMS stamps enabled intimate contact with the oxide surfaces. In this work, the mixing ratio of the PDMS precursor was further modified to enhance the adhesive properties, demonstrating excellent contact characteristics with a relatively rough Ga_2_O_3_ surface. The elastomeric PDMS stamp was placed on the surface of the Galinstan film without applying any external pressure and was then detached. In this process, the transparent Ga_2_O_3_ layer was successfully transferred onto the stamp. The transferred Ga_2_O_3_ film was slightly darker than the bare PDMS substrate because small Galinstan residues in the form of islands (<10 μm) remained on the substrate, as shown in Figure 5c. However, all Galinstan residues were entirely isolated from each other and wrapped with Ga_2_O_3_, resulting in the formation of a non-metallic film. The thickness of the exfoliated Ga_2_O_3_ film was measured as ~13 nm using AFM, as shown in Figure 5b. It is worth noting that the measured thickness of the Ga_2_O_3_ film in this work is thicker than that of the single surface oxide layer of Galinstan (i.e., ~3 nm) due to further oxidation during the exfoliation process. The surface roughness and embossed features of the fabricated Galinstan film, as shown in Figure 2d, could also affect the thickness of the Ga_2_O_3_ film.

To investigate the solar-blind photodetective properties of the exfoliated Ga_2_O_3_ film in consideration of its wide bandgap (~4.9 eV) [35,36,37,38], a channel between two patterned Galinstan electrodes was bridged using Ga_2_O_3_, as shown in Figure 5a. For this work, the conductive Galinstan film, prepared on a large area (50 mm × 50 mm), was patterned by laser ablation to form a channel. The transparent Ga_2_O_3_ film, individually prepared on the PDMS stamp, was placed between the channel to complete the device structure (see Figure 5b). The output current was constantly measured at a sample bias voltage of 0.1 V. Under visible light irradiation with a halogen lamp (ranging from 350 to 900 nm), the output current only increased by ~2.7% at a high light intensity of 30 mW cm^−2^ (see Figure 5c), and no significant change in the output current was observed at lower light intensities. As the contribution of the short-wavelength region in the emission spectrum is not negligible at high intensity, a small increase in the output current could be detected at light intensities above 30 mW cm^−2^. To confirm this speculation, the output current was also measured under irradiation of UV light of a 365 nm wavelength, which was contained in the emission spectrum of the halogen lamp, and the output current in effect increased by ~14.9% at a low light intensity of 0.2 mW cm^−2^. Eventually, under deep UV irradiation (254 nm wavelength) with an extremely low light intensity of 0.1 mW cm^−2^, the output current sensitively increased by up to 15.1% (see Figure 5d). These results strongly suggest that the combination of Galinstan and its surface oxide layers can be used for sensitive solar-blind photodetectors that possess remarkable advantages, such as low-cost and easy processability under ambient conditions, and flexibility.

## 4. Conclusions

We investigated characteristics of the flexible and stretchable Galinstan electrodes under various conditions including sub-zero temperature (i.e., <0 °C) and successfully demonstrated solar-blind photodetection via the spontaneous oxidation of Galinstan. In this work, a simple and rapid method was introduced for fabricating the flexible and stretchable Galinstan electrodes with precise patterns and exfoliating the surface oxide layers to complete the device structure enabling solar-blind photodetection. A suspension consisting of Galinstan microdroplets was prepared by sonication. Thin Galinstan films with thickness less than 1 μm were uniformly deposited on flexible PDMS substrates by compression of the dried suspension of the microdroplets. The Galinstan films, deposited on a large area (50 mm × 50 mm), were sequentially patterned using a fiber laser marking machine (λ~1064 nm), and accurate and desirable features with a high resolution of 20 μm were fabricated. Although the electrical conductivity of the fabricated films was lower than that of pure Galinstan, they still possessed electrical conductivity high enough to be used as flexible and stretchable electrodes even below 0 °C. For the photoactive components, thin Ga_2_O_3_ layers, spontaneously formed on the Galinstan surfaces, were exfoliated using elastomeric PDMS stamps and successfully transferred onto the patterned Galinstan electrodes to complete the device structure for solar-blind photodetection. The solar-blind photodetectors demonstrated a distinct increase of up to ~15.1% in the output current under deep UV irradiation (254 nm wavelength) with an extremely low light intensity of 0.1 mW cm^−2^, whereas no significant change was observed under visible light irradiation. These results strongly suggest that Galinstan can be used for flexible and stretchable electrodes working under extreme conditions, and the combination with its surface oxide layer also shows great potential for sensitive solar-blind photodetectors that possess outstanding advantages, such as low-cost and easy processability under ambient conditions. We anticipate that these results will contribute to the development of flexible and stretchable electronic devices based on liquid metals, which can lead to further application of sensors under extreme conditions.

## Data Availability

Data sharing is not applicable to this article.
